# Establishment of comprehensive quality evaluation model of fresh instant rice

**DOI:** 10.29219/fnr.v63.1420

**Published:** 2019-07-22

**Authors:** Qinghong Meng, Shouwen Zhang, Song Yan, Zhihong Zhang, Liqun Wang, Yinglei Zhang, Haitao Guan

**Affiliations:** 1 College of Food Engineering, Harbin University of Commerce, Harbin, China; 2 Food Processing Institute, Heilongjiang Academy of Agricultural Sciences, Harbin, China; 3 Quality and Safety Institute of Agricultural Products, Heilongjiang Academy of Agricultural Sciences, Harbin, China

**Keywords:** Japonica rice, quality index, analytic hierarchy process, evaluation model

## Abstract

**Background:**

Although the rice processing characteristics and processing quality evaluation technologies have been studied for many years in China, there have been few reports on the evaluation index system and evaluation method of fresh instant rice processing quality.

**Objective:**

The aim of this study was to establish a comprehensive quality evaluation model of fresh instant rice to achieve the effective quantitative quality analysis.

**Methods:**

A total of 108 japonica rice varieties were selected as the test samples, followed by the production of fresh instant rice. The color value, physicochemical quality, texture characteristic, and sensory quality of fresh instant rice were systematically analyzed. Difference analysis, correlation analysis, factor analysis, and cluster analysis were used to determine the representative quality indexes of fresh instant rice. Through initialization, forward, and normalization, the standardized indexes (0≤ *X_i_* ≤1) were obtained. The discriminant matrix of the analytic hierarchy process (AHP) was used to confirm the weight (*W_i_*) of each representative quality index, establishing the comprehensive quality evaluation model.

**Results:**

The variable coefficients of 12 out of 17 quality indexes were more than 10%. There were 136 correlation coefficients, including 15 cases with a significant difference at the level of *α* = 0.05 and 52 cases with a significant difference at the level of *α* = 0.01. Five representative quality indexes of fresh instant rice were selected by factor analysis and cluster analysis, including palatability, adhesiveness, b* value, resilience, and iodine color value. And the comprehensive quality evaluation model of fresh instant rice integrating representative indexes was obtained: *Y* = 0.5650 × palatability + 0.2294 × adhesiveness + 0.0328 × resilience + 0.1175 × b* value + 0.0533 × iodine color value.

**Conclusion:**

The AHP combined with factor analysis and cluster analysis can convert a number of quality indexes into a comprehensive quality index, and thus establish the comprehensive quality evaluation model of fresh instant rice, effectively performing the quantitative quality analysis. This model not only provided a scientific basis for the accurate evaluation of fresh instant rice quality, but also laid the foundation for the formulation of fresh instant rice standards in the future.

## Popular scientific summary

- A comprehensive quality evaluation model of fresh instant rice that can effectively perform quantitative analysis was established using the factor analysis, cluster analysis, and analytic hierarchy process. This model not only provided a scientific basis for the accurate evaluation of fresh instant rice quality but also laid the foundation for the formulation of fresh instant rice standards in the future.

With the accelerating development of the Chinese food industry, instant food has become the development focus of the food industry, no matter in cereals, oils, beverage, and other aspects. However, as the staple food of the Chinese population, there are few fresh instant rice in the vast consumer market. Fresh instant rice is a kind of convenient rice food that uses rice in general sense as the final form, and its edible quality is better than that of dehydrated rice (1), not only avoiding the disadvantages of high temperature frying in instant noodles, but also satisfying the needs of healthy eating and consumption in the fast-paced life of modern people. In recent years, Japan and the European and American countries have made significant breakthroughs in the technology of fresh instant rice. Dozens of convenient rice have been developed, with improved production technology and equipment (2, 3). As far as the market is concerned, hundreds of millions of rice eaters are the target customers of nutritional fresh rice, especially the migrants in coastal open cities, travel agents, field workers, and so on. As predicted, the need for fresh instant rice is urgent in the Chinese convenience food market, and the mature market demand is about 40 billion boxes, indicating the huge market potential of fresh instant rice. The studies of fresh rice mainly concentrate on the processing technologies of fresh rice (4–7), methods of inhibiting rice retrogradation (8–10), and fresh instant rice quality (11, 12). However, there have been few studies about the quality evaluation of fresh instant rice. In this study, we used the factor analysis, cluster analysis, and analytic hierarchy process (AHP) to establish the comprehensive quality evaluation model of fresh instant rice, provided a scientific basis for the accurate evaluation of fresh instant rice quality.

The quality index of fresh instant rice is the main basis for measuring the quality of fresh instant rice, and it refers to the various physicochemical and sensory properties of rice in the process of cooking and eating, including the color, iodine color value, gelatinization degree, texture feature, sensory property, and so on (11, 12). To ensure the accuracy and comprehensiveness of analysis data, and reflect the quality characteristics of fresh instant rice, domestic and foreign literatures and relevant standards were referred (1, 8, 11, 13). The quality characteristics of fresh instant rice mainly consisted of color value, physicochemical quality, texture characteristic, and sensory quality. In this study, to establish a comprehensive quality evaluation model to achieve the effective quantitative quality analysis of fresh instant rice, we first analyzed the differences and correlations of quality indexes; then factor analysis and cluster analysis were performed to determine the representative quality indexes of fresh instant rice; AHP was used to confirm the weight (*W_i_*) of each representative quality index, establishing a comprehensive quality evaluation model. This study achieved the conversion of a number of quality indexes into one comprehensive index that indicated the quality of fresh instant rice.

## Materials and methods

### Reagents

The used analytical reagents were listed as follows. HCl was purchased from Xi'an Jinda Chemical Industry, China. Iodine was purchased from Tianjin Bodi Chemical Co. Ltd., China. Potassium iodide (KI) was purchased from Tianjin Beichen Founder Reagent Factory, China. NaOH was purchased from Tianjin Dengke Chemical Reagent Co. Ltd., China. Sulfuric acid was purchased from Tianjin Longteng Chemical Co. Ltd., China. Sodium thiosulfate was purchased from Tianjin Fuyu Fine Chemical Co. Ltd., China. TaKa-amylase was purchased from Sigma, USA.

### Materials

A total of 108 japonica rice varieties were selected as the test samples in this study, including 105 varieties of japonica rice harvested from 10 japonica rice producing areas in China in 2014–2016. The 105 japonica rice varieties consisted of 51 varieties in Heilongjiang province, 30 cases in Jilin province, 12 cases in Liaoning province, 3 cases in Ningxia province, 2 cases in Jiangsu province, 2 cases in Tianjin province, 2 cases in Xinjiang province, 1 case in Hebei province, 1 case in Shandong province, and 1 case in Yunnan province. The remaining three test samples were the Japanese variety. All the test samples were stored at refrigerators of 15°C.

### Fresh instant rice processing technology and workflow

Fresh instant rice refers to rice that has been precooked and is microwave ready. The fresh instant rice was produced as previously described (14). First, rice washing: quickly wash once using suitable amounts of cold water and hot water separately, drain and add a certain amount of boiling water according to the ratio of rice to water (1:0.6). Second, microwave steam heating (RPMW001A, SATAKE, Japan; REB-100F, HISAKA, Japan): fill the box (18 cm × 12 cm × 4 cm; customized using polypropylene/ethylene-vinyl alcohol copolymer) with rice and water (the ratio was 1:1.2), and use microwaves and steam (intensity, 40 W/g; time, 30 sec) to boil the cooking water in the box. Third, seal: add boiling water according to the ratio of rice to water (1:1.4) and stir the rice to smooth it, ensuring that the rice cannot exceed the edge of box; besides, the rice was sealed with nitrogen membrane (Guangzhou Huilin Air Separation Equipment Co. Ltd., China) by sealing machine (Shantou Xinhua Packaging Machinery factory, China). Lastly, high-pressure sterilization (Zhucheng Zhongnuo Machinery Co. Ltd., China): steps included water injection step, heating step, heat preservation step, hot water recovery step, cooling water injection step, cooling cycle step, and draining and exhaust step. Other technological parameters were listed as follows: the temperature of film sealing machine pressure was170°C, the pressure during start-up was 0.18 MPa, the sterilization temperature was 117°C, the sterilization time was 15 min, the cooling temperature was 40°C, and the cooling time was 5 min. In the germicidal pot, the sealed box was sterilized by the water bath.

### Color determination of fresh instant rice

L* value refers to lightness [0 (black)–100 (white)], and the lightness increases with the rise of value; a* value refers to the extent of red and green, with the positive number indicating the red sample and negative number indicating the green sample; b* value refers to the extent of yellow and blue, with the positive number indicating the yellow sample and negative number indicating the blue sample (15–17). The L* value, a* value, and b* value were measured and recorded using CR-400 Tristimulus colorimeter (Konica Minolta Investment Co. Ltd., China). Every fresh sample was repeatedly measured 5 times, and the averages were taken as the results (excluding maximums and minimums to reduce bias).

### Determination of physicochemical quality of fresh instant rice

#### Determination of iodine color value of fresh instant rice

A total of 5.00 g fresh instant rice was put into a 50 mL colorimetric tube. Water was added to 25 mL and the mixture was shaked at 40°C for 1 h, followed by the dilution to 50 mL. After centrifuged at 3,000 rpm for 15 min, 0.5 mL KI-I_2_ solution (0.1 mol/L) and 0.5 mL HCl (0.1 mol/L) were added to 5 mL of supernatant, followed by settling to permit. After 15 min standing, iodine color value was measured at the wavelength of 620 nm (18, 19) (CARY100; Ultraviolet-Visible Spectrophotometer, Varian, USA).

#### Determination of light transmittance of fresh instant rice

The pretreatment of fresh instant rice was performed as mentioned above. The centrifugally obtained supernatant was detected at the wavelength of 620 nm, and the distilled water was set as a blank control. The *T* value indicates the light transmittance (20).

#### Determination of gelatinization degree of fresh instant rice

The gelatinization degree of fresh instant rice was measured as previously reported (21, 22).

### Determination of texture properties of fresh instant rice

Texture characteristic is an important index to evaluate the quality of rice (23, 24). At present, the methods of studying rice texture characteristics by texture analyzer (TA.XT.PLUS; Stable Micro Systems, UK) were mainly rice cake method and tri-grain method (25–27). Tri-grain method was used in this study. The parameters of the texture analyzer (trigger force, compression strain, and compression speed) in the determination process also affected the determination results (28, 29).

After opened, the steamed rice was heated for 2 min in the microwave oven and was sealed and cooled at room temperature for 30 min. Next, the texture analyzer was used to determine the texture characteristics of fresh instant rice samples. The TPA mode was selected to test hardness, adhesiveness, springiness, cohesiveness, chewiness, and resilience. Three complete rice grains were randomly selected from different parts of the fresh instant rice samples and measured in a radial form, with the test speed as 1.00 mm/sec (30). Each sample was measured for 7 times. After excluding the maximums and minimums, the means were taken as the final results (31).

### Sensory evaluation of fresh instant rice

This experiment was performed according to GB/T 15682-2008 (Inspection of grain and oils—Method for sensory evaluation of paddy or rice cooking and eating quality). There were 20 assessors. Repeated training and screening were conducted by taking the age (25–55 years) and gender balance into consideration.

After opened, the steamed rice was heated for 2 min in the microwave oven, and the standard rice was set as control. Four samples were evaluated every time. After heating, the fresh instant rice samples were served on the sample plates, and the evaluation was conducted 2 h after the meal. The smell, appearance structure, palatability (including adhesiveness, springiness, and hardness), and taste of rice were evaluated, excluding the cold rice texture. The assessors rinsed their mouths with warm water before each evaluation. First, identification of the smell of rice. The hot rice was placed under the nasal cavity; the assessors inhaled it with force to make the air form a rapid vortex and carefully discern the smell of rice. There should be a time interval between each sample to avoid olfactory fatigue. Then the color, gloss of rice surface, and rice integrity were observed, followed by the distinguishment of the palatability and taste characteristics of rice: a little rice was taken into the mouth using chopsticks, chewed for 3–5 sec, and the adhesiveness, hardness, springiness, and taste of rice were tested using the teeth, tongue, and other sensory organs. The comparison of test and control samples was performed, and the test samples were evaluated as better (slightly, fairly, much), worse (slightly, fairly, much), and same as control. The table of sensory evaluation scores of fresh instant rice was shown in Table 1. According to the results of the sensory comprehensive scores of assessors, the average value was calculated. The individual with large evaluation error (the single item score was inconsistent with the average value or the difference was more than two levels) was discarded, and the average value was calculated again after discarding. The calculation result was reserved to two decimal places.

**Table 1:** The table of sensory evaluation scores of fresh instant rice Control sample:Sample number: No.

	Compared with the control sample						
	Worse			Control	Better		
	Much	Fairly	Slightly		Slightly	Fairly	Much
Score	−3	−2	−1	0	+1	+2	+3
Smell							
Appearance structure							
Palatability							
Taste							
Sensory comprehensive score							
Comment							

### Statistical analysis

The Excel 2016 was used for data processing. The descriptive analysis, correlation analysis, factor analysis, and cluster analysis were performed using the SPSS20.0 software. The Yaaph software was applied in the AHP.

## Results

### Difference analysis of quality indexes of fresh instant rice

The mean, SD, and variable coefficient of color value, physicochemical quality, texture property, and sensory quality of 108 fresh instant rice were subjected to difference analysis. As shown in Table 2, there were different degrees of differences in 17 quality indexes among varieties. The mean variable coefficients for sensory quality, texture property, physicochemical quality, and color value were 198.57, 18.15, 17.92, and 14.29%, respectively, indicating that different fresh instant rice samples had the most significant differences in sensory quality. The variable coefficients for smell, appearance, palatability, taste, and comprehensive evaluation in sensory quality were over 100%, with significant differences between varieties. The variable coefficients of 12 out of 17 quality indexes were more than 10%, including palatability, smell, comprehensive evaluation, taste, appearance, adhesiveness, iodine color value, b* value, chewiness, light transmittance, hardness, and resilience (in descending order).

**Table 2:** Difference analysis of 17 quality indexes

	Indexes	Minimum	Maximum	Mean	SD	Variable coefficient (%)
Color value	L* value	57.57	75.43	66.79	3.71	5.56
	a* value	−2.5	−1.71	−2.18	0.18	−8.08
	b* value	1.41	7.02	4.49	1.31	29.22
Physicochemical quality	Iodine color value	0.23	0.94	0.5	0.15	30.12
	Light transmittance (%)	37.05	91.1	55.59	10.43	18.76
	Gelatinization degree (%)	78.33	98.57	91.03	4.44	4.88
Texture property	Hardness (g)	572.91	1392.48	910.52	170.34	18.71
	Adhesiveness (g · sec)	−40.95	−5.7	−19.77	6.99	−35.35
	Springiness	0.63	0.94	0.78	0.07	9.12
	Cohesiveness	0.47	0.6	0.53	0.03	5.53
	Chewiness (g)	209.1	739.38	389.21	107.27	27.56
	Resilience	0.23	0.39	0.31	0.04	12.63
Sensory quality	Smell	−0.6	1.42	0.12	0.28	231.41
	Appearance	−0.92	2.35	0.37	0.54	147.65
	Palatability	−1.08	2.83	0.24	0.63	258.33
	Taste	−0.75	1.56	0.26	0.45	171.39
	Comprehensive evaluation	−1.17	1.75	0.33	0.6	184.09

### Correlation analysis between quality indexes of fresh instant rice

As shown in Table 3, there were 136 correlation coefficients, including 15 cases with a significant difference at the level of *α* = 0.05 and 52 cases with a significant difference at the level of *α* = 0.01. According to the correlation analysis results, there existed an independent linear correlation between the quality indexes of fresh instant rice. Among color value indexes, b* value was significantly negatively correlated with L* and a* values, with the correlation coefficients as −0.569 and −0.277, respectively. Among physicochemical quality indexes, iodine color value was significantly negatively correlated with light transmittance, with the correlation coefficient as −0.295. Among texture property indexes, there were significant positive correlations among hardness, adhesiveness, springiness, cohesiveness, and chewiness; resilience was significantly positively correlated with cohesiveness, with the correlation coefficient as 0.566. Among sensory quality indexes, there were significant positive correlations among smell, appearance, palatability, taste, and sensory comprehensive evaluation, with high correlation coefficients. It should be mentioned that the above quality indicators reflected the same aspect of a particular feature; therefore, one index can be used instead of other indicators to avoid the repeated contribution of same property to rice quality, achieving the goal of cutting indexes.

**Table 3:** The correlation analysis between quality indexes of fresh instant rice

	L* value	a* value	b* value	Iodine color value	Light transmittance	Gelatinization degree	Hardness	Adhesiveness	Springiness	Cohesiveness	Chewiness	Resilience	Smell	Appearance	Palatability	Taste	Sensory comprehensive evaluation
L* value	1.000																
a* value	0.394^**^	1.000															
b* value	−0.569^**^	−0.277^**^	1.000														
**Iodine color value**	0.070	0.099	0.134	1.000													
**Light transmittance**	−0.037	−0.110	0.071	−0.295^**^	1.000												
**Gelatinization degree**	−0.050	0.030	−0.069	0.067	−0.313^**^	1.000											
**Hardness**	0.093	0.087	−0.062	0.033	0.319^**^	−0.157	1.000										
**Adhesiveness**	0.117	0.062	−0.044	−0.057	0.207^*^	−0.069	0.405^**^	1.000									
**Springiness**	0.144	−0.005	−0.090	0.089	0.132	0.115	0.379^**^	0.373^**^	1.000								
**Cohesiveness**	0.178	−0.054	−0.088	0.058	0.228^*^	-0.211^*^	0.392^**^	0.330^**^	0.471^**^	1.000							
**Chewiness**	0.140	0.031	-0.080	0.081	0.287^**^	-0.097	0.893^**^	0.516^**^	0.695^**^	0.600^**^	1.000						
**Resilience**	0.157	−0.107	−0.105	0.044	0.158	−0.255^**^	0.044	−0.198^*^	−0.138	0.566^**^	0.029	1.000					
**Smell**	0.135	0.175	−0.064	0.251^**^	−0.213^*^	0.187	−0.195^*^	−0.188	0.066	−0.254^**^	−0.185	−0.175	1.000				
**Appearance**	0.040	0.144	−0.030	0.330^**^	−0.471^**^	0.308^**^	−0.235^*^	−0.335^**^	0.064	−0.252^**^	−0.195^*^	−0.214^*^	0.641^**^	1.000			
**Palatability**	0.096	0.097	−0.079	0.351^**^	−0.478^**^	0.375^**^	−0.307^**^	−0.244^*^	0.086	−0.192^*^	−0.220^*^	−0.171	0.638^**^	0.856^**^	1.000		
**Taste**	0.049	0.125	−0.116	0.329^**^	−0.490^**^	0.378^**^	−0.344^**^	−0.250^**^	0.090	−0.213^*^	−0.259^**^	−0.196^*^	0.666^**^	0.826^**^	0.916^**^	1.000	
**Sensory comprehensive evaluation**	0.047	0.057	−0.107	0.365^**^	−0.563^**^	0.425^**^	−0.354^**^	−0.290^**^	0.028	−0.232^*^	−0.281^**^	−0.175	0.543^**^	0.789^**^	0.898^**^	0.875^**^	1.000

* Significant difference at the level of *α* = 0.05 (2-sided);

** Significant difference at the level of *α* = 0.01 (2-sided).

Besides, the results showed that the majority of indexes in physicochemical quality, texture property, and sensory quality were also independently correlated. Iodine color value in physicochemical quality was significantly positively associated with smell, appearance, palatability, taste, and sensory comprehensive evaluation. Adhesiveness in texture property was significantly negatively correlated with appearance, palatability, taste, and sensory comprehensive evaluation. Adhesiveness reflects the adhesivity of fresh instant rice, and the higher adhesiveness leads to higher values in sensory quality. Besides, resilience in texture property was significantly negatively correlated with appearance and taste.

Collectively, there were significant correlations among these quality indexes, suggesting one representative index substitute for other related indexes.

### Screening of representative quality indexes for fresh instant rice

#### Factor analysis of quality indexes of fresh instant rice

The factor analysis of 17 quality indexes was performed. The five common factors with characteristic value *λ* > 1 were selected, with the cumulative variance contribution rate as 75.626%. The variance contribution rate of the first common factor was 28.930%, mainly representing palatability, taste, sensory comprehensive evaluation, appearance, smell, and light transmittance, and thus reflecting the sensory and physical properties of fresh instant rice. The variance contribution rate of the second common factor was 17.779%, mainly representing chewiness, springiness, adhesiveness, and hardness, and thus reflecting the texture quality of fresh instant rice. The variance contribution rate of the third common factor was 10.953%, mainly representing L* value, a* value, and b* value, and thus reflecting the color feature of fresh instant rice. The variance contribution rate of the fourth common factor was 9.580%, mainly representing cohesiveness and resilience, and thereby also reflecting the texture quality of fresh instant rice. Besides, the variance contribution rate of the fifth common factor was 8.384%, mainly consisting of iodine color value and gelatinization degree, and thereby reflecting the physicochemical quality of fresh instant rice (Table 4).

**Table 4:** Factor analysis results of 17 quality indexes

Indexes	Weight of factor				
	1	2	3	4	5
**Palatability**	0.943	−0.065	0.039	−0.018	−0.041
**Taste**	0.933	−0.090	0.051	−0.051	−0.064
**Sensory comprehensive evaluation**	0.919	−0.134	0.014	−0.012	−0.109
**Appearance**	0.890	−0.080	0.014	−0.094	0.071
**Smell**	0.690	−0.061	0.134	−0.164	0.173
**Light transmittance**	−0.574	0.235	−0.057	0.084	0.053
**Chewiness**	−0.163	0.939	0.047	0.117	0.099
**Springiness**	0.177	0.821	0.009	0.075	−0.183
**Hardness**	−0.275	0.762	0.071	0.009	0.238
**Adhesiveness**	−0.272	0.647	0.096	−0.222	−0.058
L* value	0.072	0.114	0.839	0.158	0.050
a* value	−0.061	−0.042	−0.802	−0.141	0.332
b* value	0.094	0.030	0.669	−0.317	0.368
**Resilience**	−0.171	−0.134	0.101	0.897	0.143
**Cohesiveness**	−0.153	0.562	0.062	0.707	0.032
**Iodine color value**	0.490	0.137	−0.098	0.115	0.593
**Gelatinization degree**	0.448	0.054	−0.041	−0.225	−0.553
**Characteristic value**	4.918	3.022	1.862	1.629	1.285
**Contribution rate/%**	28.930	17.779	10.953	9.580	8.384
**Cumulative contribution rate %**	28.930	46.710	57.663	67.243	75.626

#### Cluster analysis of quality indexes of fresh instant rice

There were different similarities between the quality indexes of fresh instant rice. Cluster analysis can be used to classify the indexes; it separates the indexes with large differences and gathers similar indexes together to reduce the number of indexes. We used Ward’s clustering method to screen the quality indexes of fresh instant rice. The basic idea of Ward’s method is to treat each index as one class first, and a total of *n* indexes are divided into *n* classes; the distance between every two indexes is calculated, and the two classes with the nearest distance are merged into one class; then the distances between the current *n*–1 indexes are calculated, and the two classes with the nearest distance are merged; this process is repeated until all the indexes are combined into one class.

**Fig. 1:**
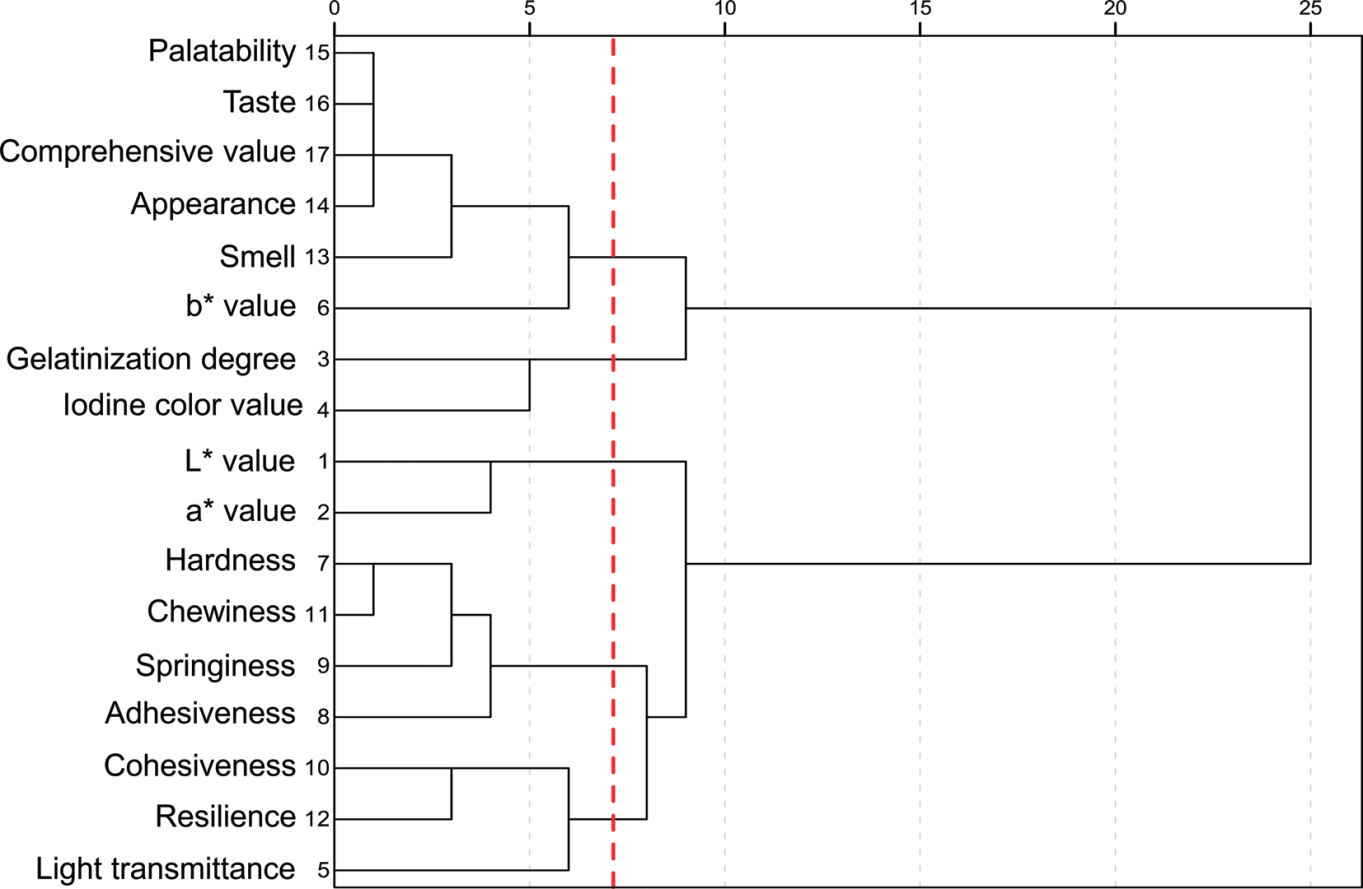
The tree-clustering diagram of quality indexes.

Ward’s clustering method classified the 17 quality indexes into 5 classes when the distance between categories was 7, obtaining the tree-clustering diagram of quality indexes (Fig. 1). The first class consisted of palatability, taste, sensory comprehensive evaluation, appearance, smell, and b* value, indicating the sensory and physical properties of fresh instant rice. The second class included gelatinization degree and iodine color value, reflecting the color and physicochemical features of fresh instant rice. The third class contained L* and a* values, suggesting the color feature of fresh instant rice. The fourth class consisted of chewiness, springiness, adhesiveness, and hardness, reflecting the texture quality of fresh instant rice. In addition, the fifth class included light transmittance, cohesiveness, and resilience, indicating the texture and physical properties of fresh instant rice.

#### Confirmation of representative quality indexes for fresh instant rice

According to the results from factor analysis and cluster analysis, the 17 quality indexes were divided into 5 classes, with consistent results. Firstly, palatability, taste, sensory comprehensive evaluation, appearance, and smell were the common indexes that belonged to the first common factor of factor analysis and the first class of cluster analysis, indicating the sensory property of fresh instant rice. The variable coefficient of palatability (258.33%) among the five indexes was the biggest, and thus palatability was selected as the representative quality index. Second, chewiness, springiness, adhesiveness, and hardness were the common indexes that belonged to the second common factor of factor analysis and the fourth class of cluster analysis, reflecting the texture quality of fresh instant rice. And the variable coefficient of adhesiveness (−35.35%) was the biggest, and thus adhesiveness was selected as the representative quality index. Third, L* and a* values were the common indexes that belonged to the third common factor of factor analysis and the third class of cluster analysis, indicating the color feature of fresh instant rice. The b* value was not included in the same class as L* and a* values by cluster analysis. However, the b* value was significantly negatively correlated with L* and a* values with the correlation coefficients as −0.569 and −0.277, respectively. Therefore, L*, a*, and b* values were allocated into one class, and the b* value with the biggest variable coefficient (29.22%) was selected as the representative quality index. Fourthly, cohesiveness and resilience were classified into the fourth common factor of factor analysis and the fifth class of cluster analysis, indicating the texture property of fresh instant rice. The variable coefficient of resilience (12.63%) was bigger, and thus resilience was selected as the representative quality index. Lastly, iodine color value and gelatinization degree were classified into the fifth common factor of factor analysis and the second class of cluster analysis, indicating the physicochemical property of fresh instant rice. Light transmittance was also included in the physicochemical property of fresh instant rice and was significantly negatively correlated with iodine color value and gelatinization degree with the correlation coefficients as −0.295 and −0.313, respectively. Therefore, the light transmittance was added to this class. Among the three indexes, the variable coefficient of iodine color value (30.12%) was the biggest, rendering iodine color value as the representative quality index. Taken together, the five representative quality indexes of fresh instant rice consisted of palatability, adhesiveness, b* value, resilience, and iodine color value.

### Establishment of comprehensive quality evaluation model of fresh instant rice

#### Forward-processing and normalization of evaluation indexes

Due to the different characteristics of the representative quality indicators screened by the comprehensive quality evaluation of fresh instant rice, it was necessary to initialize and normalize the indexes; because the indexes were positive or negative, they should be forward-processed; through mathematical transformation, each indicator was transformed into standardized data that can be calculated by the comprehensive evaluation model.

As the optimal values of the various quality indexes were different, it was necessary to determine the ideal value (*x*_0_) of each quality index (Table 5). The palatability and iodine blue values were positive indexes, with the larger measured value being the better; the resilience was neutral, with the measured value about 0.30 as the ideal value; the adhesiveness and b* value were negative indexes, with the smaller measured value being the better.

**Table 5:** The ideal value (*x*_0_) of each quality index

	Palatability	Adhesiveness (g · sec)	Resilience	b* value	Iodine blue
***x*_0_**	2.83	−40.95	0.3	1.41	0.94

Then the five quality indexes were initialized. The initialization method was the absolute value of the distance between each quality index value and the ideal value. The initialized quality indicator values ranged from *X_i_* ≥ 0. The closer the original index value was to the ideal value, the smaller the initialized value was. For the convenience of the comprehensive evaluation, these values need to be forward-processed. Meanwhile, to avoid the impact of different orders of magnitude on the comprehensive evaluation, these values need to be normalized. The pattern after forward-processing and normalization of the five quality indicators of fresh instant rice was that the closer the original index value was to the ideal value from the positive and negative directions, the closer to 1, where 0 < *X_i_* < l.

#### Determination of weights of comprehensive evaluation indexes

Different indexes have different importance to the comprehensive evaluation of fresh instant rice, and thus the *W_i_* coefficient of each representative index needs to be determined. Here, AHP was used to confirm the *W_i_* of each representative quality index. According to the contribution and importance of the five representative quality indexes to the quality of fresh instant rice, 1–9 scaling (32) was used to construct the hierarchical structure of comprehensive evaluation indexes (Table 6). The top layer was the target layer, namely, the evaluation of the comprehensive quality of fresh instant rice (A). The middle layer was the criterion layer, in which there were four elements: sensory quality (B1), texture property (B2), color feature (B3), and physicochemical quality (B4). The third layer was the scheme layer, which enumerated the possible schemes. For example, in the texture property, we constructed the third layer with adhesion (C2) and resilience (C3). The resulting discriminant matrix was shown in Table 7.

**Table 6:** Hierarchical structure of comprehensive evaluation indexes

**Comprehensive quality of fresh instant rice (A)**				
**Sensory quality** (B1)	**Texture property** (B2)		**Color feature** (B3)	**Physicochemical quality** (B4)
**Palatability** (C1)	**Adhesiveness** (C2)	**Resilience** (C3)	b* value (C4)	**Iodine color value** (C5)

**Table 7:** Discriminant matrix and its consistency check

A	B1	B2	B3	B4	B1	C1	B2	C2	C3	B3	C4	B4	C5
**B1**	1	3	5	7	C1	1	C2	1	7	C4	1	C5	1
**B2**	1/3	1	3	5			C3	1/7	1				
**B3**	1/5	1/3	1	3									
**B4**	1/7	1/5	1/3	1									
**CR = 0.0438**							CR = 0						

CR, consistency ratio.

After constructed, the discriminant matrix needed to be tested for consistency to determine whether it was applicable. If the consistency ratio (CR) was over 0.1, the consistency of the matrix was considered to pass the test; if CR > 0.1, the discriminant matrix needed to be readjusted. We found that the CRs of the discriminant matrix were less than 0.10 (0.0438, 0.0438, and 0), indicating the consistent correlations between the five representative quality indexes in the discriminant matrix. Based on the hierarchical total ordering results, the *W_i_* of palatability, adhesiveness, resilience, b* value, and iodine color value were 0.5650, 0.2294, 0.0328, 0.1175, and 0.0553, respectively. These results indicated the most significant contribution of palatability to the comprehensive quality of fresh instant rice.

#### Establishment of the evaluation model

According to the index differences, index correlations, and factor analysis, the representative quality indexes of fresh instant rice, including palatability, adhesiveness, b* value, resilience, and iodine color value, were selected. Through initialization, forward, and normalization, the representative quality indexes were converted to standardized indexes (0 ≤ *X_i_* ≤ 1). Then the discriminant matrix of AHP was used to confirm the *W_i_* of each representative quality index. The addition of standardized indexes and matched *W_i_* was used as the comprehensive evaluation value (*Y*), and the formula was listed as follows.

$$Y=\overset{n}{\underset{i=1}{\Sigma }}X_{i}\cdot W_{i}$$

Furthermore, the formulaic model integrating palatability, adhesiveness, b* value, resilience, and iodine color value (*Y* = 0.5650 × palatability +0.2294 × adhesiveness +0.0328 × resilience +0.1175 × b* value +0.0533 × iodine color value) were applied in calculating the comprehensive evaluation values of fresh instant rice varieties.

#### Verification test

The nine samples of the ‘Sino-Japanese Excellent Indica Rice Variety Breeding and Taste Tasting Symposium’ were selected as verification test samples. The results of the tasting were shown in Table 8 (33). The tasting results were obtained by two repeating evaluations by 25 expert assessors from both China and Japan.

**Table 8:** Verification of the results of Sino-Japanese appreciation of the test samples

Ranking	Sample name
**1**	Koshihikari (Uonuma)
**2**	Jijing 511
**3**	Jingyou 653
**4**	Hinohikari (Hiroshima)
**5**	Nanjing 46
**6**	Jinchuan No.1
**7**	Kenxiangdao 10179
**8**	Yanjing 219
**9**	Ningjing 43

The representative indexes of the nine samples, including palatability, adhesiveness, b* value, resilience, and iodine color value, were determined, followed by the initiation, forward-processing, and normalization of these indexes. The standardized indexes were substituted into the above comprehensive evaluation model of the fresh instant rice, and the comprehensive evaluation value Y was obtained. The measurement results of representative indexes and the comprehensive evaluation values of the nine samples were shown in Table 9.

**Table 9:** The measurement results of representative indexes and the *Y* comprehensive evaluation values of the nine test samples

Sample name	Palatability	Adhesiveness (g · sec)	Resilience	b* value	Iodine blue	*Y* comprehensive evaluation value
**Kenxiangdao 10179**	0.61	−9.34	0.26	2.65	0.83	0.42
**Jijing 511**	1.94	−22.62	0.27	4.75	0.52	0.64
**Jingyou 653**	1.58	−17.42	0.28	4.36	0.51	0.56
**Nanjing 46**	0.70	−23.18	0.30	3.41	0.43	0.49
**Ningjing 43**	−0.58	−8.54	0.26	5.39	0.65	0.17
**Yanjing 219**	−0.42	−19.85	0.33	6.19	0.48	0.25
**Jinchuan No.1**	0.60	−21.39	0.28	5.13	0.52	0.43
**Koshihikari (Uonuma)**	2.38	−36.31	0.25	4.69	0.57	0.79
**Hinohikari (Hiroshima)**	1.41	−30.05	0.26	5.67	0.61	0.59

According to the comprehensive evaluation value *Y* in Table 9, it can be concluded that the order of quality of the nine verified experimental samples was as follows: Koshihikari (Uonuma), Jijing 511, Hinohikari (Hiroshima), Jingyou 653, Nanjing 46, Jinchuan No.1, Kenxiangdao 10179, Yanjing 219, and Ningjing 43. These results were basically consistent with the results of the Sino-Japanese tasting in Table 8. Only the sorting of the Jingyou 653 and the Hinohikari (Hiroshima) was interchanged (the high adhesiveness of the Hinohikari resulted in an increase in the *Y*). These results indicate that the comprehensive evaluation model of fresh instant rice quality has high accuracy.

#### Distribution of comprehensive evaluation values of fresh instant rice

**Fig. 2:**
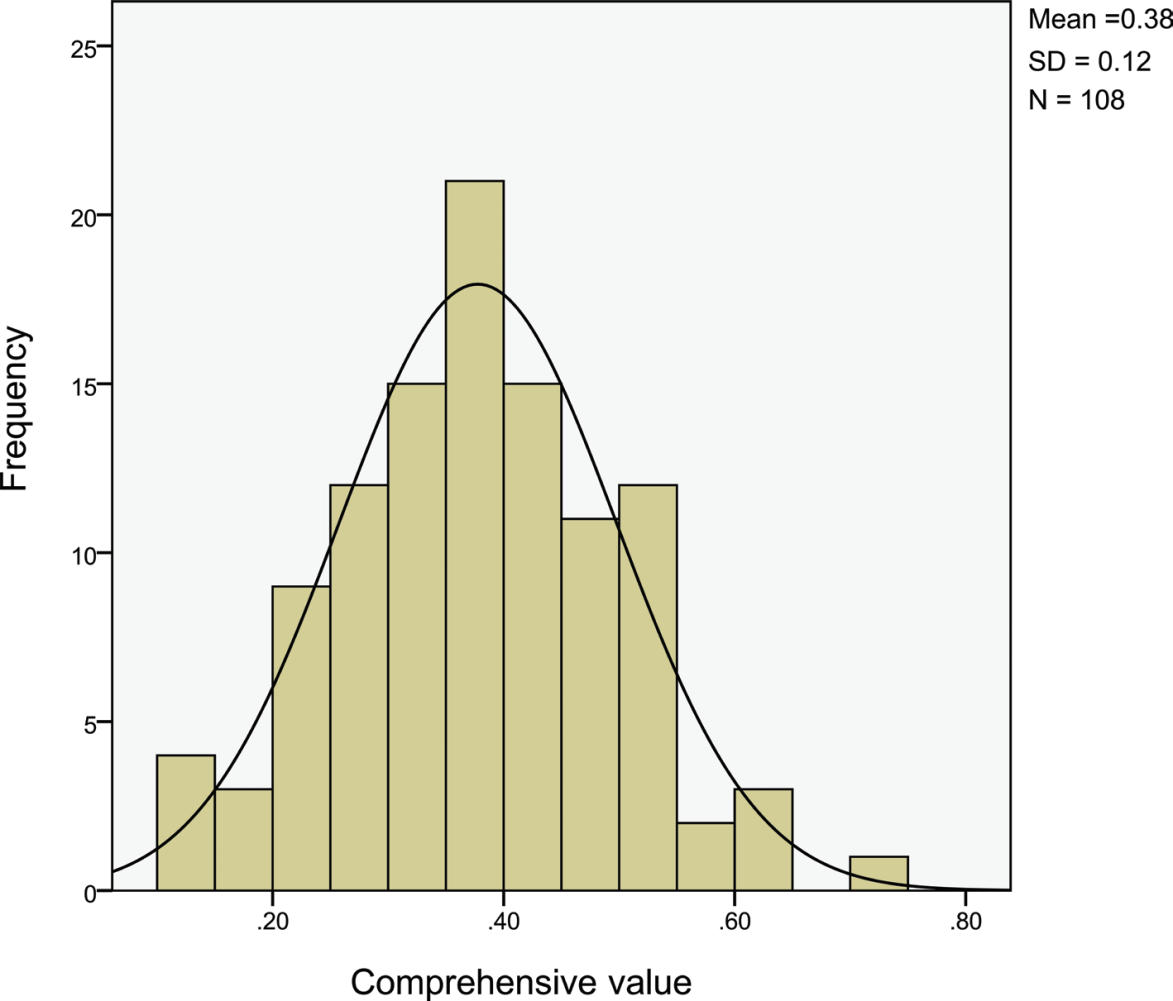
The distribution of comprehensive values.

As shown in Table 10, the mean of comprehensive evaluation values of 108 fresh instant rice varieties was 0.378, and variable coefficient was 31.788%. The distribution graph showed that the comprehensive values were mainly distributed between 0.3 and 0.5, including 26 varieties between 0.4 and 0.5 and 36 varieties between 0.3 and 0.4 (Fig. 2). Besides, the number of varieties with comprehensive values ≥0.7, 0.6≤ *Y* <0.7, 0.5 ≤ *Y* <0.6, 0.2≤ *Y* <0.3, and 0.1≤ *Y* <0.2 was 1, 3, 14, 21, and 7, respectively.

**Table 10:** The distribution of comprehensive evaluation values

	N	Minimum	Maximum	Mean	SD	Variable coefficient (%)	Skewness	Kurtosis
**Comprehensive value**	108	0.120	0.750	0.378	0.120	31.788	0.132	0.118

## Discussion

The rice processing characteristics and processing quality evaluation technologies have been studied for many years in China. However, there have been few reports on the evaluation index system and evaluation method of fresh instant rice processing quality. It is of great significance to establish a scientific comprehensive quality evaluation model of fresh instant rice. There are relative independences and certain correlations between the 17 quality indexes. It is difficult to judge the quality of fresh instant rice of different varieties by single index or multiple indexes. Moreover, the measurement of a large number of indexes is quite complicated, so it is necessary to select the representative evaluation indexes (34). In this study, the 17 quality indexes of the fresh instant rice were measured. The descriptive analysis showed that there were 12 out of the 17 indicators with a variable coefficient exceeding 10%, from high to low: palatability, smell, sensory comprehensive score, taste, appearance, adhesiveness, iodine blue value, b* value, chewiness, light transmittance, hardness, and resilience. Correlation analysis showed that there were 136 correlation coefficients, including 15 cases with significant difference at the level of *α* = 0.05 and 52 cases with significant difference at the level of *α* = 0.01. Moreover, there were significant correlations among these quality indexes, and one representative index can be used to substitute for other related indexes.

Factor analysis is a statistical method to extract common factors from variable groups and also a way to reduce the dimension in the multivariate statistical analysis (35). It focuses on how to convert many original variables into a few factor variables with the least amount of information loss and making the factor variable being strongly interpretable (36, 37). By factor analysis, multiple single indexes can be transferred to a few comprehensive indexes that cover the main information, reducing the phase differences and dimensions of data sets, realizing the reduction of data sets, and thus achieving the index simplification. Cluster analysis is a multivariate statistical analysis method to explore the correlation between indicators through a large symmetric matrix, and the result of the analysis is a cluster. It quantitatively determines the affinities between indexes. After taking the relationship between multiple factors and dominant role into consideration, the indexes are divided into different classes according to the varying degrees of affinities, achieving dimension reduction, and thus making the classification more objective to reflect the inherent connection between things. The combination of factor analysis and cluster analysis could better screen the indexes on the basis of preserving raw data information (38–40), thereby providing convenience for original information (41). In this study, using factor analysis and cluster analysis, combined with the variable coefficients and correlation analysis of quality indexes, five representative quality indexes (palatability, adhesiveness, b* value, resilience, and iodine color value) of fresh rice were screened out among 17 quality indexes, achieving the scientific classification of quality indexes of fresh instant rice.

AHP is a qualitative, quantitative, systematic, and hierarchical decision analysis method of multiobjective complex problems. It is particularly applicable to problems that are difficult to quantify completely, rationalizing the problem scientifically at a simpler level than the original one, thus avoiding the bias caused by subjective judgment. AHP is an effective method to evaluate the *W_i_* of indexes and has been used in the quality evaluation of apple (32), white wine (42), and steamed bun (43). In this study, AHP was used to confirm the *W_i_* of each representative quality index, quantifying the effect of each index on fresh instant rice quality. AHP is a semiquantitative method, which combines human factors and mathematical calculation. The discriminant matrix is established on the basis of the requirements for product quality, and subjective experience of decision-makers plays an important role. Therefore, it should be comprehensive and objective in building a discriminant matrix, which can be verified and adjusted according to the results of the consistency check. The analytical results were obtained by a mathematical operation, and the analytic hierarchy score was used as the comprehensive evaluation value of fresh rice samples, and thus the comprehensive evaluation model of fresh rice quality was obtained. This model integrating five representative quality indexes (palatability, adhesiveness, b* value, resilience, and iodine color value) could be applied in calculating the comprehensive evaluation values of 108 fresh instant rice varieties. The verification test proved that the AHP was more operative and practical, with higher accuracy.

## Conclusions

The AHP combined with factor analysis and cluster analysis can convert a number of quality indexes into a comprehensive quality index, and thus establish the comprehensive quality evaluation model of fresh instant rice (*Y* = 0.5650 × palatability + 0.2294 × adhesiveness + 0.0328 × resilience + 0.1175 × b* value + 0.0533 × iodine color value), enabling the effective performance of the quantitative quality analysis. This model not only provided a scientific basis for the accurate evaluation of fresh instant rice quality, but also laid the foundation for the formulation of fresh instant rice standards in the future.
